# Adipose tissue is associated with kidney function parameters

**DOI:** 10.1038/s41598-023-36390-z

**Published:** 2023-06-06

**Authors:** Katharina Mueller-Peltzer, Ricarda von Krüchten, Roberto Lorbeer, Susanne Rospleszcz, Holger Schulz, Annette Peters, Fabian Bamberg, Christopher L. Schlett, Blerim Mujaj

**Affiliations:** 1 Department of Diagnostic and Interventional Radiology, Faculty of Medicine Freiburg, Medical Center, Universitätsklinikum Freiburg, University of Freiburg, Hugstetter Str. 55, 79106 Freiburg, Germany; 2 Department of Radiology, Ludwig-Maximilians-University Hospital, Munich, Germany; 3 Chair of Epidemiology, Medical Faculty, Institute for Medical Information Processing, Biometry and Epidemiology, Ludwig-Maximilians-Universität München, Munich, Germany; 4 Institute of Epidemiology, German Research Center for Environmental Health, Helmholtz Zentrum München, Neuherberg, Germany; 5 German Center for Cardiovascular Research (DZHK), Partner Site Munich Heart Alliance, Munchen, Germany; 6 German Center for Diabetes Research, München-Neuherberg, Neuherberg, Germany; 7 General Practice, Huisartsenpraktijk, Bremtstraat 116, 9320 Aalst, Belgium

**Keywords:** Biochemistry, Nephrology

## Abstract

Obesity is characterized by the accumulation of adipose tissue in different body compartments. Whether adipose tissue directly affects kidney function is still unknown. We aimed to investigate the role of the adipose tissue and circulating creatinine, cystatin C and kidney function in subjects free of cardio-renal diseases. In the KORA-MRI population-based study, 377 subjects (mean age 56.2 ± 9.2 years; 41.6% female) underwent whole-body 3T-MRI examination. Adipose tissue defined as visceral adipose tissue (VAT) and subcutaneous adipose tissue (SAT) were quantified from T1-DIXON sequence using a semi-automatic algorithm. Serum creatinine and cystatin C were measured using standard laboratory and estimated glomerular filtration rate (e-GFR) was performed based on creatinine (e-GFR_crea_), cystatin C (e-GFR_cys_) and creatinine-cystatin C (e-GFR_cc_). Linear regression analysis, adjusted for risk factors, was used to investigate the relationship between adipose tissue and circulating creatinine, cystatin C, and kidney function. In multivariate analyses VAT was inversely associated with eGFR_cys_ (ß = − 4.88, *p* =  < 0.001), and positively associated with serum cystatin C (ß = 0.05, *p* =  < 0.001), respectively. No association was found between other adipose parameters such as total adipose tissue (TAT) and subcutaneous adipose tissue (SAT) and serum creatinine, urine microalbumin and eGFR_crea_. Stratified analyses according to BMI revealed confirmatory results for category of BMI > 30. VAT is positively associated with serum cystatin C and inversely with eGFR based on cystatin C, suggesting a direct involvement of visceral adipose tissue in increased metabolism of cystatin C and consequently decreased kidney function.

## Introduction

Obesity is a major risk factor for cardiovascular (CVD) and kidney diseases (KD) which lead to premature death worldwide^1^. Obesity is characterized by accumulation and deposition of adipose tissue in various body compartments, mainly as visceral adipose tissue (VAT) and subcutaneous adipose tissue (SAT)^2,3^. The potential mechanisms that link obesity to KD remain unclear. The biological functions of adipose tissue influence various metabolic processes, and fat mass is a strong predictor of metabolic changes that also affect renal function. Glomerular filtrate rate (GFR) is useful index of kidney function. A decrease of GFR may indicate acute kidney injury (AKI) or chronic kidney disease (CKD). GFR is estimated based on biomarkers such as serum creatinine and cystatin C^4,5^. Serum creatinine is biomarker for estimating GFR, but its use contains limitations, considering that several factors may influence serum creatinine such as muscle mass, protein- rich food, physical activity, and less sensitive to acute decrease of GFR^6,7^. On the other hand cystatin C as a non-glycosylated protein is synthesized and released by most nucleated cells at a constant rate, filtered by the glomerulus, and catabolized by proximal tubules^8^. Hence, GFR is the most important determinant of cystatin C concentration, and less affected compared to creatinine. However, it may also be influenced by factors other than GFR, including obesity, thyroid disease, inflammation, tobacco use, and steroid therapy^8–10^. Nevertheless, previous studies have reported that adipose tissue, defined as VAT and SAT, is associated with impaired renal function^11^, especially when the cystatin C equation was used^12^. A recent animal study investigated the expression of cystatin C by adipocytes and found that growth hormone (GH) and triiodothyronine (T3) increased the production of cystatin C by adipocytes in a time-dependent manner^13^. Yet, mechanisms linking adipose tissue to cystatin C in humans are unknown. Given this gap in evidence, we aimed to investigate the association of VAT and SAT with biomarkers of renal function in subjects without renal diseases in a population-based cohort KORA-MRI study.

## Material and methods

### Study population

KORA-MRI is a cross-sectional subsample (N = 400) of a longitudinal, population-based cohort (KORA FF4, N = 2279) in the Augsburg region, Germany^14^. After the regular examination at the KORA study center, participants underwent a whole-body MRI scan at 3 T between June 2013 and September 2014^14^. The following exclusion criteria were applied: Age > 74 years; participants with a known history of coronary artery disease, myocardial infarction, peripheral heart disease, stroke, and/or unavailable oral glucose tolerance test, pregnancy, poor general health, or other physical limitations. In addition, subjects with contraindications to MRI examination were excluded, such as known gadolinium contrast allergy, cardiac stents, pacemakers or implantable cardioverter-defibrillators, implanted metal devices, lactating women, subjects with claustrophobia, and subjects with impaired renal function, as defined by serum creatinine > 115 µmol/L. Finally, MRI scans were completed (n = 400), and subjects with incomplete measurements of VAT (n = 10), SAT (n = 16), cystatine C (n = 7) and urine albumin (n = 2) were further excluded (overlap cases). A total of 377 subjects were included in the current analysis. The Institutional Research Ethics Board of the Medical Faculty of Ludwig-Maximilian University Munich approved KORA-MRI, all participants gave written informed consent, and the study complied with the Helsinki declaration on human research^15^.

### Assessment of e-GFR, serum biomarkers and urine microalbumin

Estimates of GFR were calculated on the basis of serum creatinine, cystatin C and creatinine-cystatin C, according to the equations of the Chronic Kidney Disease Epidemiology Collaboration^16,17^. Venous blood was drawn from all study participants at the examination appointment and sent to the laboratory department of Augsburg Central Hospital within 2 to 4 h. Serum creatinine concentration were analyzed using an enzymatic colorimetric method (Dimension Vista 1500, Siemens Healthcare Diagnostics, Eschborn, Germany, or Cobas c702, Roche Diagnostics GmbH, Mannheim, Germany). Cystatin C levels were assessed using a nephelometric immunoassay (normal range 0.50–0.96 mg/L, Roche Diagnostics GmbH, Mannheim, Germany)^18,19^. Urinary albumin concentration was measured using an immunoturbidimetric assay (Tina-quant_Albumin in Urine, Boehringer Mannheim, Germany) from a single urine sample stored at − 80 °C^19^. eGFR was calculated on the basis of the Chronic Kidney Disease Epidemiology Collaboration equation, which is based on both serum creatinine and cystatin C^16^.

### Whole-body MR imaging

The protocol included sequences for the entire body (from the neck to below the hip) to quantify tissues/organs as well as for specific organs, e.g., brain, carotid arteries, or fat compartments. A 3 Tesla MRI scan (MagnetomSkyra, Siemens AG, Healthineers, Erlangen, Germany) with an 18-channel body surface coil and a table-mounted spine matrix coil was used for whole-body MRI imaging. A 2-point DIXON-T1 sequence with submaximal inspiratory breath-hold and an acquisition time of 15 s was used for body adipose tissue volume analysis. The slice thickness was 3 mm, acquired coronally, with a FOV of 488 × 716, a matrix of 256 × 256, a time of repetition (TR) of 4.06 ms, and a time of echo (TE) of 1.26 ms^20^. All images were evaluated by blinded, independent, and experienced readers who did not know the clinical characteristics of the study participants.

### MR-image analysis for adipose tissue depots

In a fat-selective tomogram (slice thickness 5 mm in 5 mm increments), based on the interpolated volume, adipose tissue was calculated in three-dimensional fat images from the 2-point-DIXON sequence. An in-house algorithm based on Matlab R2013a was used to semi-automatically quantify adipose tissue from cardiac apex to the femoral head, and segmentations were manually adjusted as needed^14^. VAT quantification was performed from the femoral head to the diaphragm, and SAT quantification was completed from the femoral head to the cardiac apex by each reader in the same manner. Total adipose tissue (TAT) was calculated as the sum of VAT and SAT^20^.

### Other risk factors

Physical examination, interviews, and blood sampling after overnight fasting were used to collect information on risk factors from all subjects. Height and weight were used to calculated Body mass index (BMI) and body surface area (BSA), and questionnaires were used to record smoking status, alcohol consumption (g/day), and use of glucose-lowering, diuretics, antihypertensive, and lipid-lowering (statins) medications. Physical activity was defined as being physically active if participants exercised regularly in summer and winter and were active ≥ 1 h per week in at least one season, or physically inactive if they exercised less. Diabetes mellitus was defined according to the WHO criteria as prediabetes (impaired glucose tolerance, IGT: normal fasting glucose concentration and a 2-h serum OGTT glucose concentration between 7.8 and 11.1 mmol/L; and/or an impaired fasting glucose concentration, as defined by fasting glucose levels between 6.1 and 6.9 mmol/L, and a normal 2-h serum glucose concentration), and diabetes (2 h serum glucose concentration determined by OGTT > 11.1 mmol/L and/or a fasting glucose level > 6.1 mmol/L). Glycated hemoglobin (HbA1c) analyzed in hemolyzed whole blood using the cation-exchange high-performance liquid chromatographic, photometric VARIANT-II-TURBO HbA1c Kit-2.0 assay, and on a VARIANT-II-TURBO Hemoglobin Testing System (Bio-Rad Laboratories Inc., Hercules, USA). Serum fasting glucose (FG) was sampled, and 75 g of anhydrous glucose (Dextro OGT; Boehringer Mannheim, Mannheim, Germany) was administered to participants who had no known diagnosis of type 2 diabetes or who were not taking anti-hyperglycemic medication. Serum FG was measured using an enzymatic colorimetric method (Dimension Vista 1500, Siemens Healthcare Diagnostics, Eschborn, Germany or Cobas c702, Roche Diagnostics GmbH, Mannheim, Germany). Serum fasting insulin (FI) levels were determined from blood samples using serum FI and were measured by a solid-phase enzyme-labeled chemiluminescent immunometric assay (Immulite 2000 Xpi, Siemens Healthcare Diagnostics, Eschborn, Germany) or by an electrochemiluminescence immunoassay (Cobas e 602, Roche Diagnostics GmbH, Mannheim, Germany). Hypertension was defined as systolic blood pressure > 140 mmHg, and diastolic blood pressure > 90 mmHg, or by currently taking antihypertensive medications. Total cholesterol and LDL-cholesterol were measured using the Boehringer CHOD-PAP (Roche Diagnostics) assay, and HDL-cholesterol using the phosphotungstic acid method (Boehringer Mannheim). Triglycerides were measured with the Boehringer GPO-PAP assay (non-fasting in diabetic subjects).

### Statistical analysis

The distributions of the study population characteristics for continuous and categorical variables were described by using mean and standard deviation (SD), median (interquartile ranges (IQRs)), or frequency with percentage, respectively. Differences between women and men were tested by t-test, Wilcoxon rank-sum test, or chi2-test, respectively. A natural logarithmic transformation was performed to normalize the distribution of TAT, VAT, SAT, and triglycerides. A two-step approach was used to investigate the associations of TAT, VAT, and SAT with kidney function parameters. First, using linear regression analysis we investigated the associations of TAT, VAT, and SAT with eGFR_crea_, eGFR_cys_, eGRF_cc_, serum creatinine, serum cystatin C, and urine microalbumin providing β-coefficients with 95% confidence intervals. Model 1 was adjusted for sex and age. Model 2 was adjusted for smoking, alcohol use, and physical activity, additionally. Model 3 was further adjusted for BSA, diabetes mellitus, systolic and diastolic blood pressure, and total cholesterol, while model 4 was further adjusted for antihypertensive, diuretic, lipid lowering, and anti-diabetic medication. Second, we investigated differences for sex and BMI categories (< 25, ≥ 25–30, and > 30) in stratified analysis, while adjusting for the above mentioned models. A *p* value < 0.05 was considered to indicate statistical significance. All analyses were performed using Stata (Stata 16.1 Corporation, College Station, TX, USA).

## Results

The study population characteristics are summarized in the Table 1. The mean age of the study population was 56.2 ± 9.2 years, 41.6% were women. There were 20.4% current smokers and 59.4% of participants were physically active. Also, 13.5% and 33.4% had a history of diabetes and hypertension, respectively. Eight percent took glucose lowering medication and 24.7% took hypertensive medication. The mean GFR was e-GFR_crea_ 86.8 mL/min/1.73 m^2^, e-GFR_cys_ 92.4 mL/min/1.73 m^2^ and e-GFR_cc_ 90.2 mL/min/1.73 m^2^ whereas mean serum creatinine and cystatin C was 78.1 ± 13.7 µmol/L and 0.88 ± 0.15 mg/L, respectively. 12.7% took diuretic medication. No study participant had a history of nephrolithiasis.

**Table 1:** Study population characteristics.

	All	Female	Male	*P*
	N = 377	N = 157	N = 220	
Age, years	56.2 (± 9.2)	56.2 (± 9)	56.2 (± 9.4)	0.978
Women	157 (41.6%)	157 (100%)	0 (0%)	–
BMI, kg/m^2^	27.9 (± 4.7)	27.4 (± 5.3)	28.2 (± 4.1)	0.084
Body surface area, m^2^	1.95 (± 0.21)	1.78 (± 0.16)	2.07 (± 0.16)	< 0.001
Waist circumference, cm	98 (± 13.7)	91.3 (± 13.7)	102.8 (± 11.6)	< 0.001
Hip circumference, cm	106.5 (± 8.6)	106.4 (± 10.2)	106.6 (± 7.2)	0.844
Waist-to-hip ratio	0.92 (± 0.09)	0.86 (± 0.07)	0.96 (± 0.07)	< 0.001
Smoking status				0.057
Never	140 (37.1%)	68 (43.3%)	72 (32.7%)	
Past	160 (42.4%)	56 (35.7%)	104 (47.3%)	
Current	77 (20.4%)	33 (21%)	44 (20%)	
Alcohol use, (g/day)	18.6 (± 24.1)	8.5 (± 14)	25.8 (± 27)	< 0.001
Physical activity	224 (59.4%)	101 (64.3%)	123 (55.9%)	0.101
Diabetes,	51 (13.5%)	13 (8.3%)	38 (17.3%)	0.012
HbA1c, %	5.57 (± 0.73)	5.53 (± 0.51)	5.59 (± 0.85)	0.430
Fasting glucose, mmol/L	5.50 (5.11; 6.11)	5.27 (4.88; 5.86)	5.66 (5.27; 6.27)	< 0.001
Fasting insulin, pmol/L	54.6 (37.2; 81)	49.3 (34.8; 74.9)	60 (38.3; 87.1)	0.004
Antidiabetic medication,	30 (8%)	11 (7%)	19 (8.6%)	0.564
Hypertension,	126 (33.4%)	44 (28%)	82 (37.3%)	0.061
Systolic blood pressure, mm/Hg	120.6 (± 16.4)	113.2 (± 14)	125.9 (± 16)	< 0.001
Diastolic blood pressure, mm/Hg	75.4 (± 9.9)	72.2 (± 8.5)	77.6 (± 10.2)	< 0.001
Antihypertensive medication,	93 (24.7%)	42 (26.8%)	51 (23.2%)	0.428
Total cholesterol, mmol/L	5.62 (± 0.94)	5.66 (± 0.89)	5.6 (± 0.97)	0.522
HDL, mmol/L	1.6 (± 0.46)	1.82 (± 0.46)	1.44 (± 0.38)	< 0.001
LDL, mmol/L	3.61 (± 0.84)	3.53 (± 0.82)	3.66 (± 0.86)	0.151
Triglycerides, mmol/L	1.22 (0.87; 1.77)	1.06 (0.77; 1.37)	1.43 (0.98; 2.13)	< 0.001
Lipid lowering medication	41 (10.9%)	17 (10.8%)	24 (10.9%)	0.980
Serum Creatinine, µmol/L	78.1 (± 13.7)	68.4 (± 10.6)	85.1 (± 11.3)	< 0.001
Serum Cystatin C, mg/L	0.88 (± 0.15)	0.86 (± 0.17)	0.89 (± 0.14)	0.044
eGFR crea, mL/min/1.73m^2^	86.8 (± 13)	86.1 (± 13.2)	87.4 (± 12.8)	0.347
eGFR cys, mL/min/1.73m^2^	92.4 (± 16.7)	90.8 (± 16.9)	93.6 (± 16.5)	0.112
eGFR cc, mL/min/1.73m^2^	90.2 (± 13.9)	88.6 (± 14.2)	91.3 (± 13.7)	0.071
Urine microalbumin, mg/mmol	19.9 (± 70.6)	11.3 (± 17.5)	26.1 (± 90.8)	0.044
Diuretic medication	48 (12.7%)	23 (14.7%)	25 (11.4%)	0.345
TAT, L	11.8 (8.6; 16.1)	10.6 (7.2; 16.2)	12.0 (9.1; 16.1)	0.037
VAT, L	4.04 (2.42; 6.12)	2.68 (1.32; 4.06)	5.39 (3.48; 7.28)	< 0.001
SAT, L	7.03 (5.42; 9.88)	8.33 (5.73; 11.58)	6.65 (5.36; 8.65)	< 0.001

The values represent mean ± standard deviation (SD), median (interquartile ranges) or frequency along with percentage (%). *P* = *p* value for difference based on t-test, Wilcoxon rank-sum test or chi2-test, respectively.

*BMI* body mass index, *eGFR*_*cc*_ estimated glomerular filtration rate based on serum creatinine and cystatin, *eGFR*_*crea*_ estimated glomerular filtration rate based on serum creatinine, *eGFR*_*cys*_ estimated glomerular filtration rate based on serum cystatin, *HbA1c* glycated hemoglobin A1c, *HDL* high density lipoprotein, *LDL* low density lipoprotein, *SAT* subcutaneous adipose tissue, *TAT* total adipose tissue, *VAT* visceral adipose tissue.

### Association between adipose tissue and e-GFR

**Figure 1:**
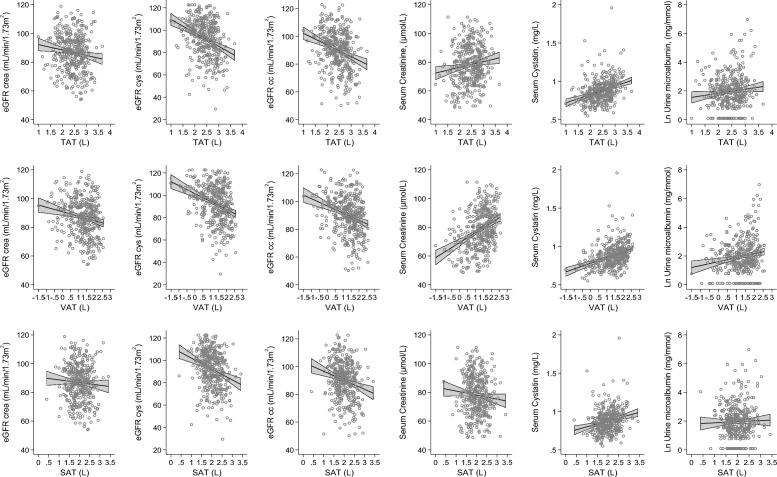
Scatter-plots Association between TAT, VAT and SAT with e-GFR, serum biomarkers and urine microalbumin. The scatter plots depicting the univariate regression line (continues line) and 95% confidence interval (outer lines with gray area). Abbreviation: eGFR_cc_ = estimated glomerular filtration rate based on serum creatinine and cystatin; eGFR_crea_ = estimated glomerular filtration rate based on serum creatinine; eGFR_cys_ = estimated glomerular filtration rate based on serum cystatin; SAT = subcutaneous adipose tissue; TAT = total adipose tissue; VAT = visceral adipose tissue.

An inverse association between VAT and e-GFR_cys_ in univariate model was found (model 1; ß = – 4.88, *p* = < 0.001) (Table 2 and Fig. 1), and with additional adjustment for risk factors in multivariate models the association remained significant (model 4; ß = − 2.77, *p* = 0.023). An inverse association was observed between TAT and SAT with e-GFR_cys_ in model 1 and 2 but was no longer significant in models 3 and 4. A similar observation could be made regarding TAT, VAT, and SAT with e-GFR_cc_ in models 1 and 2 but no more significance was reached after additional adjustment in models 3 and 4. No association was found between TAT, VAT and SAT with e-GFR_crea_.

**Table 2:** Association between TAT, VAT and SAT with serum biomarkers, e-GFR and urine microalbumin.

Per SD log transformed	Model 1	*p* value	Model 2	*p* value	Model 3	*p* value	Model 4	*p* value
eGFR crea								
TAT	− 0.62 (− 1.81; 0.57)	0.305	− 0.80 (− 2.02; 0.43)	0.204	1.49 (− 0.38; 3.36)	0.117	1.62 (− 0.3; 3.53)	0.098
VAT	− 0.94 (− 2.42; 0.53)	0.209	− 1.32 (− 2.84; 0.19)	0.086	0.36 (− 1.62; 2.33)	0.722	0.37 (− 1.65; 2.39)	0.719
SAT	− 0.48 (− 1.66; 0.71)	0.428	− 0.55 (− 1.77; 0.68)	0.380	2.06 (0.21; 3.91)	0.029	2.29 (0.4; 4.17)	0.017
eGRF cys								
TAT	− 4.07 (− 5.49; − 2.66)	< 0.001	− 4.09 (− 5.54; − 2.64)	< 0.001	− 2.52 (− 4.73; − 0.31)	0.025	− 2.18 (− 4.45; 0.09)	0.06
VAT	− 4.88 (− 6.63; − 3.12)	< 0.001	− 4.71 (− 6.51; − 2.91)	< 0.001	− 3.07 (− 5.39; − 0.75)	0.01	− 2.77 (− 5.15; − 0.39)	0.023
SAT	− 3.77 (− 5.19; − 2.36)	< 0.001	− 3.84 (− 5.28; − 2.39)	< 0.001	− 1.84 (− 4.04; 0.36)	0.102	− 1.41 (− 3.65; 0.83)	0.217
eGFR cc								
TAT	− 2.65 (− 3.84; − 1.46)	< 0.001	− 2.75 (− 4.00; − 1.51)	< 0.001	− 0.58 (− 2.48; 1.31)	0.546	− 0.34 (− 2.27; 1.6)	0.734
VAT	− 3.32 (− 4.80; − 1.85)	< 0.001	− 3.44 (− 4.97; − 1.90)	< 0.001	− 1.59 (− 3.58; 0.4)	0.117	− 1.44 (− 3.47; 0.6)	0.166
SAT	− 2.39 (− 3.58; − 1.20)	< 0.001	− 2.47 (− 3.71; − 1.23)	< 0.001	0.14 (− 1.74; 2.03)	0.881	0.5 (− 1.41; 2.41)	0.609
Serum creatinine								
TAT	0.54 (− 0.61; 1.68)	0.357	0.71 (− 0.47; 1.89)	0.236	− 1.64 (− 3.43; 0.14)	0.071	− 1.78 (− 3.62; 0.05)	0.057
VAT	0.78 (− 0.64; 2.19)	0.283	1.17 (− 0.29; 2.62)	0.116	− 0.59 (− 2.48; 1.30)	0.542	− 0.6 (− 2.54; 1.33)	0.54
SAT	0.43 (− 0.70; 1.57)	0.453	0.50 (− 0.68; 1.67)	0.405	− 2.15 (− 3.92; − 0.37)	0.018	− 2.39 (− 4.19; − 0.59)	0.009
Serum cystatin								
TAT	0.04 (0.02; 0.05)	< 0.001	0.04 (0.02; 0.05)	< 0.001	0.02 (0; 0.05)	0.048	0.02 (0; 0.04)	0.088
VAT	0.05 (0.03; 0.07)	< 0.001	0.05 (0.03; 0.06)	< 0.001	0.03 (0.01; 0.06)	0.011	0.03 (0; 0.05)	0.02
SAT	0.04 (0.02; 0.05)	< 0.001	0.04 (0.02; 0.05)	< 0.001	0.02 (− 0.01; 0.04)	0.182	0.01 (− 0.01; 0.04)	0.317
Urine microalbumin								
TAT	9.01 (1.74; 16.28)	0.015	8.08 (0.49; 15.67)	0.037	7.89 (− 3.42; 19.20)	0.171	9.38 (− 2.07; 20.82)	0.108
VAT	9.53 (0.50; 18.55)	0.039	8.16 (− 1.23; 17.54)	0.088	4.23 (− 7.70; 16.15)	0.486	6.18 (− 5.88; 18.25)	0.314
SAT	8.30 (1.07; 15.53)	0.025	7.55 (0.01; 15.09)	0.050	8.89 (− 2.34; 20.12)	0.120	9.57 (− 1.71; 20.86)	0.096

The beta estimate given with a 95% confidence interval represents the estimate size between TAT, VAT and SAT with Kidney function parameters from linear regression models. Model 1 = adjusted for sex and age; Model 2 = model 1 + smoking, alcohol use, physical activity; Model 3 = model 2 + BSA, diabetes mellitus, systolic and diastolic blood pressure, total cholesterol; Model 4 = model 3 + antihypertensive medication, diuretic medication, lipid lowering medication and anti-diabetic medication.

*SAT* subcutaneous adipose tissue, *TAT* total adipose tissue, *VAT* visceral adipose tissue.

### Association between adipose tissue and serum biomarkers

In univariate and multivariate models a positive association was found between VAT and serum cystatin C (model 4; ß = 0.03, *p* = 0.02) (Table 2). A positive association between TAT and SAT with serum cystatin C was found in models 1 and 2 but was no longer significant in models 3 and 4. No association was found between TAT, VAT and SAT with serum creatinine.

### Association between adipose tissue and urine microalbumin

Despite an initial significance for the association between TAT, VAT and SAT with urine microalbumin in univariate model (Table 2), the association was no longer significant after additional adjustment in multivariate models.

### Association between adipose tissue and e-GFR, serum biomarkers, and urine microalbumin according to BMI categories and gender

Applying BMI categories we found an inverse association between VAT and e-GFR_cys_ and a positive association between VAT and serum cystatin C in the category of BMI > 30, no similar results were found in the other BMI categories (Table S3). No association was found for other adipose tissue volumes and e-GFR, serum biomarkers, and urine microalbumin. Furthermore, when were stratified by sex, we observed an inverse association between VAT and e-GFR_cys_ only in females (Table S4). We did not observe any further differences for the association between adipose tissue depots and e-GFR, serum biomarkers, or urine microalbumin.

## Discussion

In the population-based cohort of subjects without a history of renal disease, we found a positive association between visceral adipose tissue and serum cystatin C and an inverse association with e-GFR_cys_.

Previous studies in humans has demonstrated evidence linking adipose tissue and serum cystatin C^12,21^. Cystatin C is a basic protein released at a constant rate by most cells and excreted from the bloodstream by the kidneys and is an excellent indicator of glomerular filtration rate. Cystatin C is considered superior to creatinine as a marker for detection of mild renal impairment^22^. Moreover, differences in tissue origin and production rates of the two compounds exist. The muscular source of creatinine is well documented, but the relative contribution of different organs to circulating Cystatin C levels is unknown and is difficult to estimate. In this context our results provide new insights into the relationship between adipose tissue and elevated serum cystatin C and further contribute to understanding of adipose tissue activity and cystatin C. Our findings are in line with an animal study which assessed the role of adipose tissue in increased production of cystatin C in mice^13^.

Previous studies have reported an association of adipose tissue with renal function, mainly based on traditional fat measurements such as waist-to-hip ratio, BMI and other anthropometric measurements^11,23^. Alternatively, in the Framingham Offspring Study participants underwent abdominal computed tomography scans for VAT and SAT quantification, and neither VAT nor SAT were related with an increased odds ratio for chronic kidney disease using the eGFR_crea_, while VAT and SAT were associated with reduced eGFR_cys_^12^. When considering VAT, results are in line with the Framingham study observing that higher VAT was linked to reduced eGFR_cys_. Traditionally, VAT has been reported to have higher pathogenicity compared to SAT regarding metabolic, cardiovascular or kidney injury risk^24–27^. VAT accumulation seems to cause intra-renal pressure leading to compression of capillaries and Henle loop, consequently reduced intrarenal blood circulation, RAAS activation and sodium reabsorption^28–30^. Initiation of sodium reabsorption and glomerular hyperfiltration cascade prone kidneys to inflammation and kidney disease. Moreover, the impact of VAT significantly increases with increased BMI characterized by accumulated abdominal fat. Obesity has also been linked to an accelerated progression of CKD in patients with pre-existing CKD^31^. Furthermore, we found corroborate results when considering the BMI categories: a BMI > 30 was associated with reduced e-GFR. Yet, the distribution of adipose tissue differs according to sex and in our study we found surprisingly an association between VAT and eGRF_cys_ in woman but not in men, despite men may have higher presence of VAT^32^. Although the sex homones play important role on adipose tissue accumulation in woman estrogen level decline exposes them to higher risk kindey diseases, also^33^. Interestingly, VAT was not associated with creatinine or eGRF_crea_. Since creatinine is a breakdown product of muscle mass and protein metabolism, adipose tissue biological activity of does not appear to be related to creatinine. However, adipose tissue is directly linked to cystatin C^34^. The adipose tissue activity is influenced by growth hormone (GH) and triiodthyronine (T3), thus production of cystatin C is enhanced by adipose tissue^12^. Over the past decade, studies on cystatin C have shown a significant association with measures of GFR, which is associated much more strongly with poor outcomes than creatinine. The use of cystatin C as a clinical marker of renal function, given its relationship to adipose tissue, raises additional questions regarding the correct assessment of eGFR in healthy subjects and patients with renal disease, particularly in the obese individuals.

One strength of the current study is the implementation of advanced 3T whole-body MRI technology with detailed protocol, included three-dimensional fat images from the 2-point-DIXON sequences, enabling detailed characterization and quantification of adipose tissue. The study recruited healthy individuals without renal disease. To our knowledge, this is first study to explore the relationship between adipose tissue, kidney function and serum Cystatin C in a population without renal function impairment. Moreover, multilevel testing was applied to confirm our results through confirmation in BMI categories. Nevertheless, the study encounters some limitations that need to be mentioned: First, the adipose tissue quantification did not include adipose tissue volumes in thorax, thighs or legs. Second, due to the cross-sectional design of the study, causal relationships cannot be established, and the results require further confirmation in different study designs and study populations. Third, our study was limited to a European population, and generalizability of results to other populations or geographic regions may be limited. Finally, we lacked information on GH and T3 hormones to exclude a possible confounding or influence of these hormones on our results.

## Conclusions

Visceral adipose tissue is positively associated with serum cystatin C and inversely associated with eGFR_cc_, suggesting a direct involvement of visceral adipose tissue in increased metabolism of cystatin C and consequently in decreased kidney function. Irrespective of confounders, excessive VAT may increase the risk for chronic kidney disease (CKD) in subjects with BMI > 30, while accurate characterization and quantification of adipose tissue volumes may provide additional information for optimized risk stratification in the general population. Further longitudinal studies are warranted to confirm our findings.

## Supplementary Information

Supplementary Information.

## Data Availability

The data are subject to national data protection laws and restrictions were imposed by the Ethics Committee of the Bavarian Medical Association to ensure data privacy of the study participants and therefore data cannot be made freely available in a public repository. Data are third party and belong to the KORA research platform, but can be accessed for specific research projects through individual project agreements. Interested researchers can request data from KORA via the KORA.passt online tool (https://epi.helmholtz-muenchen.de/). In a data request, one has to briefly describe the intended scientific question and then select the variables of interest within the KORA.passt tool. We confirm, that interested researchers, who agree on the general terms and conditions of the KORA data user agreement can access the data of KORA in the same way we did.

## References

[1] Bikbov B, Purcell CA, Levey AS, Smith M, Abdoli A, Abebe M (2020). Global, regional, and national burden of chronic kidney disease, 1990–2017: A systematic analysis for the global burden of disease study 2017. Lancet.

[2] Chen G-C, Arthur R, Iyengar NM, Kamensky V, Xue X, Wassertheil-Smoller S (2019). Association between regional body fat and cardiovascular disease risk among postmenopausal women with normal body mass index. Eur. Heart J..

[3] Fox CS, Massaro JM, Hoffmann U, Pou KM, Maurovich-Horvat P, Liu C-Y (2007). Abdominal visceral and subcutaneous adipose tissue compartments. Circulation.

[4] Inker LA, Eneanya ND, Coresh J, Tighiouart H, Wang D, Sang Y (2021). New creatinine- and cystatin C-based equations to estimate GFR without race. N. Engl. J. Med..

[5] Pottel H, Björk J, Rule AD, Ebert N, Eriksen BO, Dubourg L (2023). Cystatin C-based equation to estimate GFR without the inclusion of race and sex. N. Engl. J. Med..

[6] Shemesh O, Golbetz H, Kriss JP, Myers BD (1985). Limitations of creatinine as a filtration marker in glomerulopathic patients. Kidney Int..

[7] Delanaye P, Mariat C (2013). The applicability of eGFR equations to different populations. Nat. Rev. Nephrol..

[8] Abrahamson M, Olafsson I, Palsdottir A, Ulvsbäck M, Lundwall Å, Jensson O (1990). Structure and expression of the human cystatin C gene. Biochem. J..

[9] Zou LX, Sun L, Nicholas SB, Lu Y, Sinha S, Hua R (2020). Comparison of bias and accuracy using cystatin C and creatinine in CKD-EPI equations for GFR estimation. Eur. J. Intern. Med..

[10] Stevens LA, Schmid CH, Greene T, Li L, Beck GJ, Joffe MM (2009). Factors other than glomerular filtration rate affect serum cystatin C levels. Kidney Int..

[11] Pinto-Sietsma S-J, Navis G, Janssen WMT, de Zeeuw D, Gans ROB, de Jong PE (2003). A central body fat distribution is related to renal function impairment, even in lean subjects. Am. J. Kidney Dis..

[12] Young JA, Hwang S-J, Sarnak MJ, Hoffmann U, Massaro JM, Levy D (2008). Association of visceral and subcutaneous adiposity with kidney function. Clin. J. Am. Soc. Nephrol..

[13] Schmid C, Ghirlanda C, Zwimpfer C, Tschopp O, Zuellig RA, Niessen M (2019). Cystatin C in adipose tissue and stimulation of its production by growth hormone and triiodothyronine in 3T3-L1 cells. Mol. Cell. Endocrinol..

[14] Bamberg F, Hetterich H, Rospleszcz S, Lorbeer R, Auweter SD, Schlett CL (2017). Subclinical disease burden as assessed by whole-body MRI in subjects with prediabetes, subjects with diabetes, and normal control subjects from the general population: The KORA-MRI study. Diabetes.

[15] Association WM (2013). World medical association declaration of helsinki: Ethical principles for medical research involving human subjects. JAMA.

[16] Inker LA, Schmid CH, Tighiouart H, Eckfeldt JH, Feldman HI, Greene T (2012). Estimating glomerular filtration rate from serum creatinine and cystatin C. N. Engl. J. Med..

[17] Mujaj B, Yang W-Y, Zhang Z-Y, Wei F-F, Thijs L, Verhamme P (2019). Renal function in relation to low-level environmental lead exposure. Nephrol. Dial. Transpl..

[18] Steubl D, Block M, Herbst V, Nockher WA, Schlumberger W, Satanovskij R (2016). Plasma uromodulin correlates with kidney function and identifies early stages in chronic kidney disease patients. Medicine (Baltimore).

[19] Then C, Then HL, Lechner A, Thorand B, Meisinger C, Heier M (2021). Serum uromodulin and decline of kidney function in older participants of the population-based KORA F4/FF4 study. Clin. Kidney J..

[20] Rospleszcz S, Lorbeer R, Storz C, Schlett CL, Meisinger C, Thorand B (2019). Association of longitudinal risk profile trajectory clusters with adipose tissue depots measured by magnetic resonance imaging. Sci. Rep..

[21] Naour N, Rouault C, Fellahi S, Lavoie M-E, Poitou C, Keophiphath M (2010). Cathepsins in human obesity: Changes in energy balance predominantly affect cathepsin s in adipose tissue and in circulation. J. Clin. Endocrinol. Metab..

[22] Chew JS, Saleem M, Florkowski CM, George PM (2008). Cystatin C–a paradigm of evidence based laboratory medicine. Clin. Biochem. Rev..

[23] Gelber RP, Kurth T, Kausz AT, Manson JE, Buring JE, Levey AS (2005). Association between body mass index and CKD in apparently healthy men. Am. J. Kidney Dis..

[24] Neeland IJ, Ayers CR, Rohatgi AK, Turer AT, Berry JD, Das SR (2013). Associations of visceral and abdominal subcutaneous adipose tissue with markers of cardiac and metabolic risk in obese adults. Obesity (Silver Spring).

[25] Fox CS, Massaro JM, Hoffmann U, Pou KM, Maurovich-Horvat P, Liu CY (2007). Abdominal visceral and subcutaneous adipose tissue compartments: Association with metabolic risk factors in the Framingham Heart Study. Circulation.

[26] von Krüchten R, Lorbeer R, Müller-Peltzer K, Rospleszcz S, Storz C, Askani E (2022). Association between adipose tissue depots and dyslipidemia: The KORA-MRI population-based study. Nutrients.

[27] Kataoka H, Nitta K, Hoshino J (2023). Visceral fat and attribute-based medicine in chronic kidney disease. Front. Endocrinol..

[28] Hall JE, do Carmo JM, da Silva AA, Wang Z, Hall ME (2019). Obesity, kidney dysfunction and hypertension: Mechanistic links. Nat. Rev. Nephrol..

[29] Neeland IJ, Ross R, Després J-P, Matsuzawa Y, Yamashita S, Shai I (2019). Visceral and ectopic fat, atherosclerosis, and cardiometabolic disease: A position statement. Lancet Diabetes Endocrinol..

[30] da Silva AA, do Carmo JM, Li X, Wang Z, Mouton AJ, Hall JE (2020). Role of hyperinsulinemia and insulin resistance in hypertension: Metabolic syndrome revisited. Can. J. Cardiol..

[31] Hall ME, do Carmo JM, da Silva AA, Juncos LA, Wang Z, Hall JE (2014). Obesity, hypertension, and chronic kidney disease. Int. J. Nephrol. Renovasc. Dis..

[32] Shi H, Strader AD, Woods SC, Seeley RJ (2007). The effect of fat removal on glucose tolerance is depot specific in male and female mice. Am. J. Physiol.-Endocrinol. Metab..

[33] Demerath EW, Sun SS, Rogers N, Lee M, Reed D, Choh AC (2007). Anatomical patterning of visceral adipose tissue: Race, sex, and age variation. Obesity.

[34] Naour N, Fellahi S, Renucci JF, Poitou C, Rouault C, Basdevant A (2009). Potential contribution of adipose tissue to elevated serum cystatin C in human obesity. Obesity (Silver Spring).

