# Supplementary material for: Adipose tissue is associated with kidney function parameters
Source: Sci Rep. 2023 Jun 6;13:9151. doi: 10.1038/s41598-023-36390-z (PMC10244418; doi:10.1038/s41598-023-36390-z)
Supplement: Supplementary file 1 — Supplementary Information. [file 41598_2023_36390_MOESM1_ESM.docx]

**Supplementary Material.**

**S1** Association between TAT, VAT and SAT with serum biomarkers, e-GFR and urine microalbumin according to BMI categories

| **Per SD log transformed** | **Model 1** | **p-value** | | | | **Model 2** | | | | **p-value** | | | | **Model 3** | | | | **p-value** | | | | **Model 4** | | | **p-value** | | |  |
| --- | --- | --- | --- | --- | --- | --- | --- | --- | --- | --- | --- | --- | --- | --- | --- | --- | --- | --- | --- | --- | --- | --- | --- | --- | --- | --- | --- | --- |
| **BMI <25 (N=104)** | **eGFR crea** | | | | | | | | | | | | | | | | | | | | | | | | | | | |
| TAT | 0.8 (-2.82; 4.42) | 0.663 | | | | 0.63 (-3.04; 4.31) | | | | 0.734 | | | | 3.39 (-1.22; 8.00) | | | | 0.148 | | | | 2.83 (-1.78; 7.44) | | | 0.226 | | |  |
| VAT | 1.02 (-2.94; 4.98) | 0.61 | | | | 0.37 (-3.7; 4.44) | | | | 0.857 | | | | 1.83 (-2.89; 6.54) | | | | 0.444 | | | | 1.64 (-3.03; 6.32) | | | 0.487 | | |  |
| SAT | 0.15 (-3.47; 3.77) | 0.935 | | | | 0.27 (-3.35; 3.89) | | | | 0.882 | | | | 2.97 (-1.64; 7.57) | | | | 0.204 | | | | 2.55 (-2.03; 7.12) | | | 0.272 | | |  |
|  | **eGRF cys** | | | | | | | | | | | | | | | | | | | | | | | | | | | |
| TAT | 0.08 (-4.19; 4.35) | | 0.972 | | | | 0.54 (-3.69; 4.76) | | | | 0.802 | | | | 1.22 (-4.08; 6.52) | | | | 0.649 | | | | 1.20 (-3.97; 6.37) | | | 0.646 | | |
| VAT | -1.71 (-6.36; 2.95) | | 0.469 | | | | -0.5 (-5.17; 4.18) | | | | 0.833 | | | | -0.93 (-6.31; 4.46) | | | | 0.733 | | | | -0.55 (-5.78; 4.67) | | | 0.833 | | |
| SAT | 0.59 (-3.67; 4.85) | | 0.785 | | | | 0.56 (-3.59; 4.72) | | | | 0.789 | | | | 1.30 (-3.99; 6.58) | | | | 0.627 | | | | 1.43 (-3.70; 6.55) | | | 0.581 | | |
|  | **eGFR cc** | | | | | | | | | | | | | | | | | | | | | | | | | | | |
| TAT | 0.43 (-3.28; 4.15) | | | 0.819 | | | | 0.64 (-3.18; 4.46) | | | | 0.74 | | | | 2.49 (-2.31; 7.30) | | | | 0.306 | | | | 2.19 (-2.52; 6.90) | | | 0.359 | |
| VAT | -0.66 (-4.72; 3.4) | | | 0.746 | | | | -0.27 (-4.5; 3.97) | | | | 0.901 | | | | 0.19 (-4.72; 5.10) | | | | 0.940 | | | | 0.30 (-4.48; 5.07) | | | 0.902 | |
| SAT | 0.49 (-3.21; 4.2) | | | 0.792 | | | | 0.56 (-3.2; 4.32) | | | | 0.768 | | | | 2.48 (-2.31; 7.27) | | | | 0.307 | | | | 2.33 (-2.34; 6.99) | | | 0.324 | |
|  | **Serum Creatinine** | | | | | | | | | | | | | | | | | | | | | | | | | | | |
| TAT | -0.88 (-4.27; 2.51) | | | | 0.606 | | | | -0.76 (-4.18; 2.66) | | | | 0.66 | | | | -3.27 (-7.56; 1.01) | | | | 0.132 | | | -2.68 (-6.92; 1.55) | | | 0.211 | |
| VAT | -1.49 (-5.19; 2.21) | | | | 0.425 | | | | -0.92 (-4.7; 2.86) | | | | 0.631 | | | | -2.25 (-6.63; 2.12) | | | | 0.309 | | | -2.07 (-6.36; 2.22) | | | 0.340 | |
| SAT | -0.08 (-3.47; 3.31) | | | | 0.963 | | | | -0.23 (-3.6; 3.14) | | | | 0.892 | | | | -2.68 (-6.96; 1.60) | | | | 0.217 | | | -2.22 (-6.43; 1.99) | | | 0.297 | |
|  | **Serum Cystatin** | | | | | | | | | | | | | | | | | | | | | | | | | | | |
| TAT | 0 (-0.05; 0.04) | | | | 0.912 | | | | -0.01 (-0.05; 0.04) | | | | 0.729 | | | | -0.01 (-0.06; 0.04) | | | | 0.709 | | | -0.01 (-0.06; 0.04) | | | 0.710 | |
| VAT | 0.02 (-0.03; 0.06) | | | | 0.49 | | | | 0 (-0.04; 0.05) | | | | 0.864 | | | | 0.01 (-0.04; 0.07) | | | | 0.657 | | | 0.01 (-0.04; 0.06) | | | 0.751 | |
| SAT | -0.01 (-0.05; 0.03) | | | | 0.681 | | | | -0.01 (-0.05; 0.03) | | | | 0.667 | | | | -0.01 (-0.07; 0.04) | | | | 0.601 | | | -0.02 (-0.07; 0.04) | | | 0.554 | |
|  | **Urine Microalbumin** | | | | | | | | | | | | | | | | | | | | | | | | | | | |
| TAT | -1.58 (-6.75; 3.59) | | | | 0.546 | | | | -0.38 (-5.52; 4.77) | | | | 0.885 | | | | 1.04 (-4.80; 6.88) | | | | 0.724 | | | 0.36 (-4.62; 5.34) | | | 0.885 | |
| VAT | 1.09 (-4.57; 6.74) | | | | 0.703 | | | | 2.37 (-3.3; 8.05) | | | | 0.409 | | | | 2.72 (-3.18; 8.63) | | | | 0.362 | | | 1.73 (-3.28; 6.74) | | | 0.495 | |
| SAT | -2.52 (-7.66; 2.62) | | | | 0.334 | | | | -1.66 (-6.71; 3.4) | | | | 0.517 | | | | 0.92 (-4.90; 6.74) | | | | 0.754 | | | -0.08 (-5.02; 4.85) | | | 0.974 | |

The beta estimate given with a 95% confidence interval represents the estimate size between TAT, VAT and SAT with Kidney function parameters from linear regression models. Model 1 = adjusted for sex and age; Model 2 = model 1 + smoking, alcohol use, physical activity; Model 3 = model 2 + BMI, diabetes mellitus, systolic and diastolic blood pressure, total cholesterol, history of nephrolithiasis; Model 4 = model 3 + antihypertensive medication, diuretic medication, lipid lowering medication and antidiabetic medication. Abbreviation: SAT = subcutaneous adipose tissue; TAT = total adipose tissue; VAT = visceral adipose tissue.

**S2** Association between TAT, VAT and SAT with serum biomarkers, e-GFR and urine microalbumin according to BMI categories

| **Per SD log transformed** | **Model 1** | **p-value** | | | | **Model 2** | | | | **p-value** | | | | **Model 3** | | | | **p-value** | | | | **Model 4** | | | **p-value** | | |  |
| --- | --- | --- | --- | --- | --- | --- | --- | --- | --- | --- | --- | --- | --- | --- | --- | --- | --- | --- | --- | --- | --- | --- | --- | --- | --- | --- | --- | --- |
| **BMI >25 – 30 (N=165)** | **eGFR crea** | | | | | | | | | | | | | | | | | | | | | | | | | | | |
| TAT | 0.66 (-2.51; 3.82) | 0.682 | | | | 0.47 (-2.75; 3.68) | | | | 0.774 | | | | 4.37 (0.71; 8.04) | | | | 0.020 | | | | 4.62 (0.86; 8.38) | | | 0.016 | | |  |
| VAT | 0.2 (-2.74; 3.14) | 0.893 | | | | -0.3 (-3.32; 2.72) | | | | 0.845 | | | | 2.37 (-0.91; 5.65) | | | | 0.156 | | | | 2.48 (-0.91; 5.87) | | | 0.150 | | |  |
| SAT | 1.63 (-1.83; 5.09) | 0.354 | | | | 1.8 (-1.71; 5.31) | | | | 0.311 | | | | 5.93 (2.05; 9.81) | | | | 0.003 | | | | 6.18 (2.25; 10.12) | | | 0.002 | | |  |
|  | **eGRF cys** | | | | | | | | | | | | | | | | | | | | | | | | | | | |
| TAT | -3.69 (-7.23; -0.15) | | 0.041 | | | | -4.11 (-7.64; -0.59) | | | | 0.023 | | | | -3.48 (-7.74; 0.79) | | | | 0.109 | | | | -3.29 (-7.49; 0.91) | | | 0.124 | | |
| VAT | -2.38 (-5.69; 0.93) | | 0.158 | | | | -2.49 (-5.83; 0.86) | | | | 0.144 | | | | -1.64 (-5.44; 2.16) | | | | 0.395 | | | | -1.45 (-5.21; 2.30) | | | 0.446 | | |
| SAT | -3.61 (-7.5; 0.28) | | 0.069 | | | | -4.12 (-7.99; -0.26) | | | | 0.037 | | | | -3.15 (-7.73; 1.43) | | | | 0.176 | | | | -2.83 (-7.29; 1.63) | | | 0.212 | | |
|  | **eGFR cc** | | | | | | | | | | | | | | | | | | | | | | | | | | | |
| TAT | -1.77 (-4.8; 1.27) | | | 0.252 | | | | -2.1 (-5.18; 0.98) | | | | 0.179 | | | | 0.44 (-3.2; 4.08) | | | | 0.81 | | | | 0.65 (-2.99; 4.28) | | | 0.726 | |
| VAT | -1.24 (-4.06; 1.58) | | | 0.386 | | | | -1.58 (-4.48; 1.32) | | | | 0.283 | | | | 0.4 (-2.83; 3.62) | | | | 0.808 | | | | 0.52 (-2.71; 3.75) | | | 0.75 | |
| SAT | -1.25 (-4.58; 2.08) | | | 0.46 | | | | -1.43 (-4.82; 1.95) | | | | 0.403 | | | | 1.41 (-2.48; 5.3) | | | | 0.474 | | | | 1.7 (-2.13; 5.54) | | | 0.382 | |
|  | **Serum Creatinine** | | | | | | | | | | | | | | | | | | | | | | | | | | | |
| TAT | -0.63 (-3.77; 2.51) | | | | 0.691 | | | | -0.39 (-3.59; 2.8) | | | | 0.807 | | | | -4.22 (-7.86; -0.58) | | | | 0.023 | | | -4.35 (-8.08; -0.61) | | | 0.023 | |
| VAT | -0.06 (-2.97; 2.86) | | | | 0.968 | | | | 0.48 (-2.52; 3.48) | | | | 0.751 | | | | -2.10 (-5.36; 1.17) | | | | 0.206 | | | -2.07 (-5.43; 1.29) | | | 0.226 | |
| SAT | -1.56 (-4.99; 1.87) | | | | 0.371 | | | | -1.69 (-5.17; 1.79) | | | | 0.339 | | | | -5.79 (-9.64; -1.94) | | | | 0.003 | | | -5.97 (-9.87; -2.06) | | | 0.003 | |
|  | **Serum Cystatin** | | | | | | | | | | | | | | | | | | | | | | | | | | | |
| TAT | 0.04 (0; 0.07) | | | | 0.027 | | | | 0.04 (0.01; 0.07) | | | | 0.014 | | | | 0.03 (-0.01; 0.07) | | | | 0.099 | | | 0.03 (-0.01; 0.07) | | | 0.113 | |
| VAT | 0.03 (-0.01; 0.06) | | | | 0.109 | | | | 0.03 (0; 0.06) | | | | 0.094 | | | | 0.02 (-0.02; 0.05) | | | | 0.335 | | | 0.02 (-0.02; 0.05) | | | 0.385 | |
| SAT | 0.04 (0; 0.07) | | | | 0.048 | | | | 0.04 (0.01; 0.08) | | | | 0.026 | | | | 0.03 (-0.01; 0.07) | | | | 0.169 | | | 0.03 (-0.01; 0.07) | | | 0.209 | |
|  | **Urine Microalbumin** | | | | | | | | | | | | | | | | | | | | | | | | | | | |
| TAT | 6.78 (-4; 17.56) | | | | 0.216 | | | | 7.16 (-3.85; 18.17) | | | | 0.201 | | | | 15.53 (2.74; 28.32) | | | | 0.018 | | | 12.54 (-0.52; 25.61) | | | 0.060 | |
| VAT | 7.54 (-2.44; 17.53) | | | | 0.138 | | | | 8.55 (-1.76; 18.86) | | | | 0.103 | | | | 14.08 (2.76; 25.41) | | | | 0.015 | | | 11.28 (-0.34; 22.9) | | | 0.057 | |
| SAT | -1.24 (-13.1; 10.63) | | | | 0.837 | | | | -1.26 (-13.37; 10.84) | | | | 0.837 | | | | 5.55 (-8.38; 19.47) | | | | 0.432 | | | 3.02 (-10.97; 17.01) | | | 0.670 | |

The beta estimate given with a 95% confidence interval represents the estimate size between TAT, VAT and SAT with Kidney function parameters from linear regression models. Model 1 = adjusted for sex and age; Model 2 = model 1 + smoking, alcohol use, physical activity; Model 3 = model 2 + BMI, diabetes mellitus, systolic and diastolic blood pressure, total cholesterol, history of nephrolithiasis; Model 4 = model 3 + antihypertensive medication, diuretic medication, lipid lowering medication and antidiabetic medication. Abbreviation: SAT = subcutaneous adipose tissue; TAT = total adipose tissue; VAT = visceral adipose tissue.

**S3** Association between TAT, VAT and SAT with serum biomarkers, e-GFR and urine microalbumin according to BMI categories

| **Per SD log transformed** | **Model 1** | **p-value** | | | | **Model 2** | | | | **p-value** | | | | **Model 3** | | | | **p-value** | | | | **Model 4** | | | **p-value** | | |  |
| --- | --- | --- | --- | --- | --- | --- | --- | --- | --- | --- | --- | --- | --- | --- | --- | --- | --- | --- | --- | --- | --- | --- | --- | --- | --- | --- | --- | --- |
| **BMI >30 (N=108)** | **eGFR crea** | | | | | | | | | | | | | | | | | | | | | | | | | | | |
| TAT | -0.5 (-4.51; 3.51) | 0.806 | | | | -0.88 (-5.19; 3.43) | | | | 0.686 | | | | -2.29 (-7.44; 2.86) | | | | 0.38 | | | | -1.92 (-7.2; 3.37) | | | 0.473 | | |  |
| VAT | -2.76 (-7.39; 1.86) | 0.239 | | | | -3.3 (-8.24; 1.63) | | | | 0.187 | | | | -4.37 (-9.57; 0.82) | | | | 0.098 | | | | -4.18 (-9.44; 1.09) | | | 0.118 | | |  |
| SAT | 0.57 (-2.85; 3.98) | 0.743 | | | | 0.38 (-3.3; 4.07) | | | | 0.837 | | | | -0.14 (-4.6; 4.32) | | | | 0.95 | | | | 0.52 (-4.07; 5.11) | | | 0.823 | | |  |
|  | **eGRF cys** | | | | | | | | | | | | | | | | | | | | | | | | | | | |
| TAT | -8.94 (-13.82; -4.07) | | <0.001 | | | | -6.73 (-11.8; -1.65) | | | | 0.01 | | | | -3.48 (-9.36; 2.39) | | | | 0.242 | | | | -3.11 (-9.18; 2.97) | | | 0.312 | | |
| VAT | -10.35 (-16.02; -4.69) | | <0.001 | | | | -8.94 (-14.73; -3.14) | | | | 0.003 | | | | -7.38 (-13.22; -1.55) | | | | 0.014 | | | | -6.91 (-12.89; -0.93) | | | 0.024 | | |
| SAT | -5.5 (-9.77; -1.22) | | 0.012 | | | | -3.62 (-8.04; 0.81) | | | | 0.108 | | | | 0.16 (-4.94; 5.26) | | | | 0.95 | | | | 0.58 (-4.71; 5.87) | | | 0.828 | | |
|  | **eGFR cc** | | | | | | | | | | | | | | | | | | | | | | | | | | | |
| TAT | -5.14 (-9.12; -1.16) | | | 0.012 | | | | -4.03 (-8.29; 0.22) | | | | 0.063 | | | | -2.89 (-7.96; 2.18) | | | | 0.261 | | | | -2.47 (-7.7; 2.76) | | | 0.350 | |
| VAT | -7.21 (-11.77; -2.66) | | | 0.002 | | | | -6.67 (-11.49; -1.84) | | | | 0.007 | | | | -6.36 (-11.39; -1.33) | | | | 0.014 | | | | -6.00 (-11.13; -0.86) | | | 0.023 | |
| SAT | -2.7 (-6.15; 0.76) | | | 0.125 | | | | -1.67 (-5.35; 2.02) | | | | 0.372 | | | | 0.23 (-4.17; 4.63) | | | | 0.918 | | | | 0.82 (-3.73; 5.37) | | | 0.722 | |
|  | **Serum Creatinine** | | | | | | | | | | | | | | | | | | | | | | | | | | | |
| TAT | 0.74 (-3.04; 4.52) | | | | 0.697 | | | | 1.19 (-2.87; 5.25) | | | | 0.561 | | | | 2.05 (-2.8; 6.91) | | | | 0.403 | | | 1.59 (-3.4; 6.59) | | | 0.528 | |
| VAT | 2.75 (-1.61; 7.11) | | | | 0.213 | | | | 3.38 (-1.27; 8.02) | | | | 0.152 | | | | 4.16 (-0.72; 9.05) | | | | 0.094 | | | 3.85 (-1.13; 8.82) | | | 0.128 | |
| SAT | -0.31 (-3.53; 2.91) | | | | 0.848 | | | | -0.12 (-3.59; 3.36) | | | | 0.947 | | | | -0.08 (-4.28; 4.12) | | | | 0.968 | | | -0.74 (-5.08; 3.60) | | | 0.736 | |
|  | **Serum Cystatin** | | | | | | | | | | | | | | | | | | | | | | | | | | | |
| TAT | 0.09 (0.03; 0.15) | | | | 0.002 | | | | 0.06 (0.01; 0.12) | | | | 0.033 | | | | 0.03 (-0.04; 0.1) | | | | 0.434 | | | 0.02 (-0.05; 0.10) | | | 0.507 | |
| VAT | 0.1 (0.04; 0.17) | | | | 0.002 | | | | 0.09 (0.02; 0.16) | | | | 0.01 | | | | 0.07 (0; 0.14) | | | | 0.037 | | | 0.07 (0.00; 0.14) | | | 0.052 | |
| SAT | 0.06 (0.01; 0.11) | | | | 0.024 | | | | 0.04 (-0.02; 0.09) | | | | 0.176 | | | | -0.01 (-0.07; 0.05) | | | | 0.818 | | | -0.01 (-0.07; 0.05) | | | 0.728 | |
|  | **Urine Microalbumin** | | | | | | | | | | | | | | | | | | | | | | | | | | | |
| TAT | 38.12 (-5.12; 81.36) | | | | 0.083 | | | | 32.2 (-14.19; 78.58) | | | | 0.172 | | | | 27.77 (-24.66; 80.19) | | | | 0.296 | | | 41.9 (-9.2; 93.01) | | | 0.107 | |
| VAT | 31.4 (-19.18; 81.98) | | | | 0.221 | | | | 22.95 (-30.92; 76.82) | | | | 0.4 | | | | 7.83 (-45.86; 61.53) | | | | 0.773 | | | 18.93 (-33.1; 70.96) | | | 0.472 | |
| SAT | 29.39 (-7.54; 66.32) | | | | 0.118 | | | | 27.78 (-11.83; 67.4) | | | | 0.167 | | | | 29.16 (-15.92; 74.24) | | | | 0.202 | | | 40.78 (-3.33; 84.89) | | | 0.07 | |

The beta estimate given with a 95% confidence interval represents the estimate size between TAT, VAT and SAT with Kidney function parameters from linear regression models. Model 1 = adjusted for sex and age; Model 2 = model 1 + smoking, alcohol use, physical activity; Model 3 = model 2 + BMI, diabetes mellitus, systolic and diastolic blood pressure, total cholesterol, history of nephrolithiasis; Model 4 = model 3 + antihypertensive medication, diuretic medication, lipid lowering medication and antidiabetic medication. Abbreviation: SAT = subcutaneous adipose tissue; TAT = total adipose tissue; VAT = visceral adipose tissue.

**S4** Association between TAT, VAT and SAT with serum biomarkers, e-GFR and urine microalbumin according to sex

| **Per SD log transformed** | **Model 1** | **p-value** | | | | **Model 2** | | | | **p-value** | | | | **Model 3** | | | | **p-value** | | | | **Model 4** | | | **p-value** | | |  |
| --- | --- | --- | --- | --- | --- | --- | --- | --- | --- | --- | --- | --- | --- | --- | --- | --- | --- | --- | --- | --- | --- | --- | --- | --- | --- | --- | --- | --- |
| **Female** | **eGFR crea** | | | | | | | | | | | | | | | | | | | | | | | | | | | |
| TAT | -1.86 (-3.65; -0.07) | 0.042 | | | | -1.94 (-3.83; -0.05) | | | | 0.044 | | | | 0.54 (-2.71; 3.78) | | | | 0.745 | | | | 0.43 (-2.89; 3.74) | | | 0.799 | | |  |
| VAT | -2.63 (-4.73; -0.53) | 0.015 | | | | -2.73 (-4.94; -0.52) | | | | 0.016 | | | | -0.84 (-3.97; 2.29) | | | | 0.595 | | | | -0.92 (-4.13; 2.28) | | | 0.57 | | |  |
| SAT | -1.58 (-3.42; 0.25) | 0.090 | | | | -1.67 (-3.61; 0.26) | | | | 0.090 | | | | 1.27 (-2.02; 4.55) | | | | 0.447 | | | | 1.29 (-2.02; 4.59) | | | 0.443 | | |  |
|  | **eGRF cys** | | | | | | | | | | | | | | | | | | | | | | | | | | | |
| TAT | -4.97 (-7.07; -2.87) | | <0.001 | | | | -5.24 (-7.38; -3.09) | | | | <0.001 | | | | -2.67 (-6.38; 1.03) | | | | 0.156 | | | | -2.37 (-6.21; 1.47) | | | 0.225 | | |
| VAT | -6.26 (-8.71; -3.8) | | <0.001 | | | | -6.3 (-8.82; -3.78) | | | | <0.001 | | | | -4.09 (-7.62; -0.55) | | | | 0.024 | | | | -3.94 (-7.62; -0.26) | | | 0.036 | | |
| SAT | -4.58 (-6.75; -2.41) | | <0.001 | | | | -4.92 (-7.14; -2.7) | | | | <0.001 | | | | -1.46 (-5.23; 2.31) | | | | 0.445 | | | | -1.08 (-4.94; 2.77) | | | 0.58 | | |
|  | **eGFR cc** | | | | | | | | | | | | | | | | | | | | | | | | | | | |
| TAT | -3.86 (-5.66; -2.06) | | | <0.001 | | | | -4.06 (-5.95; -2.16) | | | | <0.001 | | | | -1.13 (-4.38; 2.13) | | | | 0.494 | | | | -1.03 (-4.37; 2.31) | | | 0.544 | |
| VAT | -5.05 (-7.15; -2.96) | | | <0.001 | | | | -5.14 (-7.34; -2.93) | | | | <0.001 | | | | -2.82 (-5.93; 0.29) | | | | 0.076 | | | | -2.8 (-6.01; 0.41) | | | 0.087 | |
| SAT | -3.48 (-5.34; -1.62) | | | <0.001 | | | | -3.72 (-5.68; -1.77) | | | | <0.001 | | | | 0 (-3.31; 3.3) | | | | 0.998 | | | | 0.21 (-3.13; 3.56) | | | 0.9 | |
|  | **Serum Creatinine** | | | | | | | | | | | | | | | | | | | | | | | | | | | |
| TAT | 1.72 (0.18; 3.26) | | | | 0.029 | | | | 1.8 (0.18; 3.43) | | | | 0.030 | | | | -0.34 (-3.14; 2.46) | | | | 0.813 | | | -0.29 (-3.13; 2.55) | | | 0.841 | |
| VAT | 2.41 (0.61; 4.21) | | | | 0.009 | | | | 2.54 (0.64; 4.44) | | | | 0.009 | | | | 0.9 (-1.79; 3.6) | | | | 0.509 | | | 0.92 (-1.83; 3.66) | | | 0.51 | |
| SAT | 1.46 (-0.11; 3.04) | | | | 0.069 | | | | 1.54 (-0.13; 3.21) | | | | 0.070 | | | | -1.05 (-3.89; 1.78) | | | | 0.464 | | | -1.11 (-3.94; 1.72) | | | 0.439 | |
|  | **Serum Cystatin** | | | | | | | | | | | | | | | | | | | | | | | | | | | |
| TAT | 0.05 (0.03; 0.07) | | | | <0.001 | | | | 0.05 (0.03; 0.08) | | | | <0.001 | | | | 0.02 (-0.02; 0.06) | | | | 0.368 | | | 0.02 (-0.03; 0.06) | | | 0.413 | |
| VAT | 0.06 (0.04; 0.09) | | | | <0.001 | | | | 0.07 (0.04; 0.09) | | | | <0.001 | | | | 0.04 (0; 0.08) | | | | 0.057 | | | 0.04 (0; 0.08) | | | 0.061 | |
| SAT | 0.05 (0.02; 0.07) | | | | <0.001 | | | | 0.05 (0.03; 0.08) | | | | <0.001 | | | | 0.01 (-0.04; 0.05) | | | | 0.786 | | | 0 (-0.04; 0.05) | | | 0.881 | |
|  | **Urine Microalbumin** | | | | | | | | | | | | | | | | | | | | | | | | | | | |
| TAT | 0.5 (-2.07; 3.07) | | | | 0.700 | | | | 0.72 (-2.01; 3.45) | | | | 0.602 | | | | 4.46 (-0.18; 9.1) | | | | 0.059 | | | 3.84 (-0.91; 8.59) | | | 0.112 | |
| VAT | 1.81 (-1.21; 4.83) | | | | 0.239 | | | | 2.24 (-0.95; 5.43) | | | | 0.168 | | | | 6.61 (2.22; 11.01) | | | | 0.003 | | | 6.19 (1.66; 10.72) | | | 0.008 | |
| SAT | -0.03 (-2.66; 2.59) | | | | 0.982 | | | | 0.1 (-2.69; 2.89) | | | | 0.944 | | | | 2.2 (-2.55; 6.94) | | | | 0.362 | | | 1.61 (-3.18; 6.39) | | | 0.507 | |

The beta estimate given with a 95% confidence interval represents the estimate size between TAT, VAT and SAT with Kidney function parameters from linear regression models. Model 1 = adjusted for sex and age; Model 2 = model 1 + smoking, alcohol use, physical activity; Model 3 = model 2 + BMI, diabetes mellitus, systolic and diastolic blood pressure, total cholesterol, history of nephrolithiasis; Model 4 = model 3 + antihypertensive medication, diuretic medication, lipid lowering medication and antidiabetic medication. Abbreviation: SAT = subcutaneous adipose tissue; TAT = total adipose tissue; VAT = visceral adipose tissue.

**S5** Association between TAT, VAT and SAT with serum biomarkers, e-GFR and urine microalbumin according to sex

| **Per SD log transformed** | **Model 1** | **p-value** | | | | **Model 2** | | | | **p-value** | | | | **Model 3** | | | | **p-value** | | | | **Model 4** | | | **p-value** | | |  |
| --- | --- | --- | --- | --- | --- | --- | --- | --- | --- | --- | --- | --- | --- | --- | --- | --- | --- | --- | --- | --- | --- | --- | --- | --- | --- | --- | --- | --- |
| **Male** | **eGFR crea** | | | | | | | | | | | | | | | | | | | | | | | | | | | |
| TAT | 0.42 (-1.2; 2.04) | 0.607 | | | | 0.17 (-1.48; 1.82) | | | | 0.84 | | | | 2.08 (-0.24; 4.41) | | | | 0.079 | | | | 2.16 (-0.27; 4.58) | | | 0.081 | | |  |
| VAT | 0.66 (-1.54; 2.85) | 0.555 | | | | 0.01 (-2.23; 2.25) | | | | 0.994 | | | | 1.52 (-1.25; 4.29) | | | | 0.28 | | | | 1.44 (-1.4; 4.29) | | | 0.318 | | |  |
| SAT | 0.31 (-1.26; 1.88) | 0.699 | | | | 0.27 (-1.33; 1.86) | | | | 0.744 | | | | 2.28 (0.04; 4.52) | | | | 0.046 | | | | 2.46 (0.13; 4.8) | | | 0.039 | | |  |
|  | **eGRF cys** | | | | | | | | | | | | | | | | | | | | | | | | | | | |
| TAT | -3.48 (-5.43; -1.53) | | 0.001 | | | | -3.39 (-5.42; -1.37) | | | | 0.001 | | | | -2.43 (-5.31; 0.45) | | | | 0.098 | | | | -1.95 (-4.94; 1.03) | | | 0.198 | | |
| VAT | -4.09 (-6.75; -1.42) | | 0.003 | | | | -3.93 (-6.7; -1.16) | | | | 0.006 | | | | -2.74 (-6.16; 0.67) | | | | 0.115 | | | | -2.36 (-5.84; 1.12) | | | 0.182 | | |
| SAT | -3.37 (-5.26; -1.49) | | 0.001 | | | | -3.31 (-5.27; -1.35) | | | | 0.001 | | | | -2.14 (-4.92; 0.65) | | | | 0.132 | | | | -1.63 (-4.52; 1.26) | | | 0.267 | | |
|  | **eGFR cc** | | | | | | | | | | | | | | | | | | | | | | | | | | | |
| TAT | -1.70 (-3.31; -0.09) | | | 0.038 | | | | -1.79 (-3.47; -0.11) | | | | 0.037 | | | | -0.17 (-2.55; 2.2) | | | | 0.885 | | | | 0.12 (-2.34; 2.57) | | | 0.926 | |
| VAT | -1.92 (-4.11; 0.26) | | | 0.084 | | | | -2.18 (-4.47; 0.1) | | | | 0.061 | | | | -0.68 (-3.49; 2.14) | | | | 0.636 | | | | -0.52 (-3.39; 2.35) | | | 0.72 | |
| SAT | -1.7 (-3.25; -0.14) | | | 0.033 | | | | -1.68 (-3.31; -0.06) | | | | 0.043 | | | | 0.11 (-2.18; 2.4) | | | | 0.923 | | | | 0.48 (-1.9; 2.86) | | | 0.691 | |
|  | **Serum Creatinine** | | | | | | | | | | | | | | | | | | | | | | | | | | | |
| TAT | -0.44 (-2.13; 1.25) | | | | 0.607 | | | | -0.16 (-1.89; 1.56) | | | | 0.852 | | | | -2.44 (-4.85; -0.02) | | | | 0.048 | | | -2.52 (-5.04; -0.01) | | | 0.049 | |
| VAT | -0.75 (-3.05; 1.54) | | | | 0.518 | | | | -0.06 (-2.4; 2.28) | | | | 0.958 | | | | -1.93 (-4.81; 0.95) | | | | 0.188 | | | -1.83 (-4.78; 1.12) | | | 0.222 | |
| SAT | -0.27 (-1.91; 1.37) | | | | 0.745 | | | | -0.21 (-1.88; 1.46) | | | | 0.803 | | | | -2.52 (-4.86; -0.19) | | | | 0.034 | | | -2.74 (-5.16; -0.31) | | | 0.027 | |
|  | **Serum Cystatin** | | | | | | | | | | | | | | | | | | | | | | | | | | | |
| TAT | 0.03 (0.01; 0.05) | | | | 0.002 | | | | 0.03 (0.01; 0.05) | | | | 0.004 | | | | 0.02 (-0.01; 0.05) | | | | 0.126 | | | 0.02 (-0.01; 0.05) | | | 0.241 | |
| VAT | 0.03 (0.01; 0.06) | | | | 0.009 | | | | 0.03 (0.01; 0.06) | | | | 0.015 | | | | 0.02 (-0.01; 0.06) | | | | 0.135 | | | 0.02 (-0.01; 0.05) | | | 0.2 | |
| SAT | 0.03 (0.01; 0.05) | | | | 0.002 | | | | 0.03 (0.01; 0.05) | | | | 0.003 | | | | 0.02 (-0.01; 0.04) | | | | 0.172 | | | 0.01 (-0.01; 0.04) | | | 0.338 | |
|  | **Urine Microalbumin** | | | | | | | | | | | | | | | | | | | | | | | | | | | |
| TAT | 18.95 (5.47; 32.44) | | | | 0.006 | | | | 17.11 (2.95; 31.27) | | | | 0.018 | | | | 11.78 (-7.82; 31.38) | | | | 0.238 | | | 14.91 (-5.02; 34.84) | | | 0.142 | |
| VAT | 23.09 (4.75; 41.44) | | | | 0.014 | | | | 20.25 (1.01; 39.49) | | | | 0.039 | | | | 7.22 (-16.07; 30.51) | | | | 0.542 | | | 10.87 (-12.45; 34.2) | | | 0.359 | |
| SAT | 17.22 (4.13; 30.32) | | | | 0.01 | | | | 15.77 (2.08; 29.46) | | | | 0.024 | | | | 13.54 (-5.37; 32.45) | | | | 0.159 | | | 14.73 (-4.52; 33.99) | | | 0.133 | |

The beta estimate given with a 95% confidence interval represents the estimate size between TAT, VAT and SAT with Kidney function parameters from linear regression models. Model 1 = adjusted for sex and age; Model 2 = model 1 + smoking, alcohol use, physical activity; Model 3 = model 2 + BMI, diabetes mellitus, systolic and diastolic blood pressure, total cholesterol, history of nephrolithiasis; Model 4 = model 3 + antihypertensive medication, diuretic medication, lipid lowering medication and antidiabetic medication. Abbreviation: SAT = subcutaneous adipose tissue; TAT = total adipose tissue; VAT = visceral adipose tissue.
